# Liquid Foods

**Published:** 1887-10-29

**Authors:** 


### Liquid Foods.

By our Special Commissioner.

That liquids alone can sustain human life for a long time is easily proved by the examples of all the babies the world has ever seen, and by most of the cases of typhoid and other continued fevers. Many persons of weak digestion are strongly tempted to gradually lessen the quantity of solids daily partaken of, and at the same time to correspondingly increase the weight and volume of liquids. As a rule that is a serious mistake, for which the weak-minded suffer in due time; and the temptation to live on liquids solely or largely should never be yielded to except under medical advice. But though that is so, on the other hand many do not take a sufficient quantity of liquid, and hence arises, among other things, habitual constipation—one of the most depressing and enfeebling of non-dangerous complaints. Perhaps, however, constipation should not be classed among non-dangerous disorders; for if certain modern theorists be correct, and auto-infection of a particular kind be possible, we cannot tell how many fatal cases of typhoid and other fevers have had a remote commencement in that affection.

Food liquids are not so numerous and of so great a variety as is desirable, but there are more of them available than are always made use of. To confine our remarks in the present article to what may be called "light-meal liquids," that is, those which are suitable for breakfast, lunch, and supper, we have three principal ones, viz., tea, coffee, and cocoa. The two former are in such universal use that nothing need be said of them, except that they should always be good of their kind; that neither nor both of them should be exclusively taken; and that a sound discretion should be employed as to the frequency and time of day for their use.

Cocoa is a far less general favourite than tea or coffee, for reasons which are not difficult to explain. Nevertheless, it is a wholesome and nutritious substance, and a very important addition to our food resources. It consists, as most people are aware, of the variously prepared seeds of the cocoa-tree—*Theobroma Cacao*. The tree is small, and grows wild in the forests of Demerara and Mexico. For the purposes of the cocoa trade it is largely cultivated in the West Indies and elsewhere. Those who really wish to understand about dietetic cocoa, and to purchase and use only the best, should know one or two facts about its preparation. There are two principal ways of preparing it: one by grinding the seeds, or nibs, with the husks; and the other by grinding them without the husks. It is hardly necessary to say that the latter process, or the crushing of the seeds alone, without the husks, gives both the richest and the finest flavoured cocoa. Of course each of the products can be honestly called "cocoa," only it is manifest that the man who crushes the husks with the nibs has more for his money than he who uses the nibs only, and consequently can sell his manufactured article at a lower price. But then it must be remembered that, like all low-priced things, the article is not so good. "Cheap and nasty" applies to cocoas as it does to doctors, druggists, and almost every other article in the market. He who wants a good article must pay a good price for it. It may be relied upon that the cocoa-nibs, without the husks, give the purest cocoa. The best chocolate is prepared exclusively from the crushed nibs.

The active principle of cocoa-seed (nibs) is *theobromine*, which resembles *theine*, the active principle of tea. But in addition to this active principle the seeds contain an enormous proportion—about half their weight—of a fatty substance, called "cocoa-butter." In the best cocoas the pure and digestible parts of this butter are retained, and hence their highly nutritive qualities. The cheaper kinds are made from the "cake" which is left after the rich butter is "expressed" from it. The great botanist Linnæus gave to cocoa the name of "theobroma"—food for the gods—showing the high appreciation he entertained of its "food" qualities. Cocoa is much less stimulating to the nervous system than tea or coffee, and also much more of a "food." For nervous persons it is highly to be recommended as a frequent substitute for those articles, especially for breakfast, lunch, and supper. Once a day is quite as often as many persons should take tea or coffee; but a well-prepared cocoa may be taken twice, or even oftener, without risk, where it agrees with the stomach. The all-important point about it is to get as perfect a preparation as possible. There are a few such in the market, and among these Van Houten's occupies a very high place. Those preparations which are mixed with maize, peas, or potato-flower, and rendered tenacious by the addition of treacle, mutton-suet, and other foreign substances, have helped to bring cocoa into bad repute, and should always be avoided.

